# True Local Recurrences after Breast Conserving Surgery have Poor Prognosis in Patients with Early Breast Cancer

**DOI:** 10.7759/cureus.541

**Published:** 2016-03-24

**Authors:** Dauren Sarsenov, Serkan Ilgun, Cetin Ordu, Gul Alco, Atilla Bozdogan, Filiz Elbuken, Kezban Nur Pilanci, Filiz Agacayak, Zeynep Erdogan, Yesim Eralp, Maktav Dincer, Vahit Ozmen

**Affiliations:** 1 General Surgery, Istanbul Florence Nightingale Hospital; 2 Medical Oncology, Gayrettepe Florence Nightingale Hospital; 3 Radiation Oncology, Gayrettepe Florence Nightingale Hospital; 4 Statistics, Istanbul Florence Nightingale Hospital; 5 Radiology, Gayrettepe Florence Nightingale Hospital; 6 Medical Oncology, Istanbul Bilim University; 7 Radiology, Istanbul Florence Nightingale Hospital; 8 Physical Therapy and Rehabilitation, Istanbul Bilim University; 9 Medical Oncology, Istanbul University; 10 Department of Surgery, Istanbul University

**Keywords:** breast cancer, ipsilateral breast tumor recurrence, breast conserving surgery

## Abstract

Background:

This study was aimed at investigating clinical and histopathologic features of ipsilateral breast tumor recurrences (IBTR) and their effects on survival after breast conservation therapy.

Methods:

1,400 patients who were treated between 1998 and 2007 and had breast-conserving surgery (BCS) for early breast cancer (cT1-2/N0-1/M0) were evaluated. Demographic and pathologic parameters, radiologic data, treatment, and follow-up related features of the patients were recorded.

Results:

53 patients (3.8%) had IBTR after BCS within a median follow-up of 70 months. The mean age was 45.7 years (range, 27-87 years), and 22 patients (41.5%) were younger than 40 years. 33 patients (62.3%) had true recurrence (TR) and 20 were classified as new primary (NP). The median time to recurrence was shorter in TR group than in NP group (37.0 (6-216) and 47.5 (11-192) months respectively; p = 0.338). Progesterone receptor positivity was significantly higher in the NP group (p = 0.005). The overall 5-year survival rate in the NP group (95.0%) was significantly higher than that of the TR group (74.7%, p < 0.033). Multivariate analysis showed that younger age (<40 years), large tumor size (>20 mm), high grade tumor and triple-negative molecular phenotype along with developing TR negatively affected overall survival (hazard ratios were 4.2 (CI 0.98-22.76), 4.6 (CI 1.07-13.03), 4.0 (CI 0.68-46.10), 6.5 (CI 0.03-0.68), and 6.5 (CI 0.02- 0.80) respectively, p < 0.05).

Conclusions:

Most of the local recurrences after BCS in our study were true recurrences, which resulted in a poorer outcome as compared to new primary tumors. Moreover, younger age (<40), large tumor size (>2 cm), high grade, triple negative phenotype, and having true recurrence were identified as independent prognostic factors with a negative impact on overall survival in this dataset of patients with recurrent breast cancer. In conjunction with a more intensive follow-up program, the role of adjuvant therapy strategies should be explored further in young patients with large and high-risk tumors to reduce the risk of TR.

## Introduction

As a result of earlier detection of cancer with the wide use of mammographic screening and increased awareness of breast cancer, the number of patients who undergo breast-conserving surgery (BCS) has significantly increased, yielding comparable outcomes to mastectomy [1-4]. Local recurrence rates have substantially decreased since modern radiotherapy techniques with boost treatment and new chemotherapeutic agents combined with BCS, substantiating the role of BCS as a valid alternative to mastectomy [3, 5-8]. However, presence of positive surgical margin, lymphovascular invasion (LVI), extensive intraductal component (EIC), young age at first presentation, and high histologic grade (HG) are still known to be prognostic factors that increase the risk of local recurrence and consequently require more intensive pre-operative radiologic studies to select patients for BCS [9-14].

The incidence of local recurrences that can be classified as true recurrences or new primary tumors is reported to be between 3-5% [10, 15-16]. The histopathology of true recurrences shows features that have similarities to the primary tumor histopathology while also being close to the primary tumor bed. True recurrence (TR) rates are reported to be around 62% of ipsilateral breast tumor recurrences (IBTR) [17]. New primary (NP) tumors are classified as IBTR which are localized beyond the primary tumor’s site and have different histopathologic characteristics. NP tumors were shown to have a better prognosis with a 5-year survival of around 91%, whereas while TR tumors had a 76% 5-year survival [3, 17-19].

The aim of this study was to analyze the clinical data of patients who developed local recurrence after BCS, the clinical characteristics related to TR and NP, as well as the prognostic factors that had an impact on survival in these two distinct subgroups.

## Materials and methods

Among 1400 patients who underwent BCS with subsequent radiotherapy at the Istanbul Florence Nightingale Hospital Breast Center between 1998 and 2007, 53 patients who developed IBTR were included in the study. Patients who underwent BCS without radiotherapy, patients who had pure ductal carcinoma in situ (DCIS), patients who had neoadjuvant chemotherapy, and those who developed synchronous contralateral breast tumor recurrences or systemic metastases were excluded from the study. This study was approved by The Bilim University Ethical Committee.

Demographic data, radiologic and pathologic findings including tumor size, receptor status, histologic grade, presence of intraductal component, molecular subtypes, axillary involvement, as well as details on local and systemic treatments, and recurrence type of the 53 patients included in this data set were retrieved from patient files and evaluated for correlations.

Patients with IBTR were classified into two subgroups, the true recurrence group and the new primary group. TR was defined as recurrence in the same quadrant as the primary tumor, with histologic characteristics similar to the primary tumor. NP was defined as recurrent tumors located in a different quadrant of the breast with different histopathologic features. If a tumor had similar histopathologic properties but was located in the opposite quadrant it was accepted as new primary tumor.

Patients underwent segmental mastectomy with sentinel lymph node biopsy and/or axillary lymph node dissection if the sentinel lymph node was positive for tumor involvement. Intra-operative frozen section diagnoses with touch imprints were done with re-excisions performed when necessary. Surgical margin status was stratified into two groups: 2-5 mm and >5 mm. There was no surgical margin less than 2 mm. All patients completed their pre-planned, adjuvant radiotherapy program that mandated 50 Gy irradiation to the whole breast with a boost dose 16 Gy. Patients received systemic treatment according to the recommendations made during tumor board discussions. 24 patients were given anthracycline +/- taxane-based chemotherapy, and 3 patients received a cyclophosphamide-metotrexate-5-fluorouracil combination. Only 6 out of 10 patients who were HER-2 positive received trastuzumab in addition to conventional chemotherapeutics (mostly taxane-based regimens) because trastuzumab was not available before 2005. Hormonal treatment was given to all patients who had positive estrogen receptor (ER) and/or progesterone receptor (PR).

Molecular subtypes were defined in accordance with the St. Gallen’s surrogate definition of intrinsic subtypes of breast cancer [20]. Luminal A subtypes were ER- and PR-positive and HER-2 negative; luminal B were ER positive and/or PR positive and HER-2 negative with any Ki-67, or was HER-2 positive with low Ki-67 (under 14%). Histologic grade was used for verification when Ki-67 was not available. Patients were accepted as ER- and PR-positive if receptor expression was >1% [21]. Patients who were hormone positive and HER-2 positive were classified as HER-2 positive luminal B, whereas those who were HER-2 positive and ER/PR negative were classified as HER-2 positive. Patients who were ER/PR and HER-2 negative were categorized within the triple negative group. In cases when HER-2 was not available, re-staining and re-assessment were performed. HER-2 positive status was indicated by evidence of protein overexpression on immunohistochemical (IHC) staining or gene amplification on fluorescence in situ hybridization (FISH). IHC overexpression with a score of 3 was accepted as positive. Borderline expression of score 2 was validated using FISH.

Statistical analyses were performed using SPSS software version 17. The variables were investigated using visual (histograms, probability plots) and analytical methods (Kolmogorov-Smirnov/Shapiro-Wilk’s test) to determine whether or not they were normally distributed. Descriptive analyses were presented using means and standard deviations for normally distributed variables. Parametric variables were analyzed using one-way ANOVA test while non-parametric variables were investigated using Mann-Whitney U-test. Where appropriate, either a Chi-square test or a Fisher’s exact test (when chi-square test assumptions do not hold due to low expected cell counts) was used to assess proportions of nominal/ordinal variables in different groups. We used time to progression (relapse) as an outcome to denote recurrences from the time of initial diagnosis to the first IBTR event and did not perform PFS analysis since both NP and TR were accepted as events. The overall 5-year survival rate was calculated from the date of diagnosis to the date of last follow-up or death for any reason, using the Kaplan-Meier analysis. The univariate difference between the curves was assessed by the log rank test. Multivariate analyses were performed using proportional hazards Cox regression model which included factors such as age, type of recurrence, molecular subtypes, tumor size, and grade. A p-value of less than or equal to 0.05 was accepted as statistically significant.

## Results

53 patients (3.8%) out of 1400 patients with locally recurrent disease were selected and included in the study. The median follow-up duration was 70 months (range: 8-288 months); the median times to IBTR from initial diagnosis to occurrence of NP and TR were 37.0 (6-216 mo) and 47.5 (11-192 mo) months, respectively (p=0.338). The deaths of all patients with IBTR were related to systemic spread of disease following recurrence.

22 patients (41.5%) were aged less than 40 years. 33 patients (62.3%) were premenopausal, and 15 patients (28.3%) had a family history of breast cancer. The most frequent tumor location was the upper outer quadrant of the breast (49.1%). 33 patients (62.3%) had a TR, and 20 patients (37.7%) had a new primary tumor (Table 1).

**Table 1:** Comparison of patients’ demographic, treatment, and survival characteristics between two groups NS: Non-significant. * Median was not reached due to the low number of events in NP group.

	True Recurrence	New Primary	Total	p value
Age at initial diagnosis, %/ (n)				
≤ 40	48.5% (16)	30.0% (6)	41.5% (22)	NS
Age at local recurrence, %/ (n)				
≤ 40	39.4% (13)	10.0% (2)	28.3% (15)	p = 0.028
Family history, %/ (n)				
Present	75.8% (25)	65.0% (13)	71.7% (38)	NS
Menopausal status, %/ (n)				
Pre	63.6% (21)	60.0% (12)	62.3% (33)	NS
Systemic treatment, %/ (n)				
Received	87.9% (29)	85.0% (17)	86.8% (46)	NS
Hormonal therapy, %/ (n)				
Received	48.5% (16)	65.0% (13)	54.7% (29)	NS
Age at initial diagnosis (mean)	45.3±16.6	46.4±11.5	45.7±14.8	NS
Age at recurrence (mean)	49.7±17.1	51.7±11.3	50.5±15.1	NS
Time to recurrence (median) in months	37.0 (6-216)	47.5 (11-192)	41.0 (6-216)	NS
5 year overall survival	74.7%*	95.0%*	82.80%	p = 0.033

Sentinel lymph node biopsy (SLNB) was negative in 68% of patients, and others (32%) required axillary lymph node dissection (ALND), a primary surgery.

46 patients (86.8%) received chemotherapy followed by hormonal therapy; combined hormonal therapy alone was given to the seven remaining patients (Table 1).

There was no statistically significant difference between the two groups in terms of family history, menopausal status, age, or type of adjuvant treatment given (Table 1). Local and systemic management of IBTR was also similar among both groups. All IBTR events in patients who received trastuzumab were true local recurrences.

The distribution of pathologic features including histologic type, HG, tumor stage and nodal status, and presence of LVI and EIC are given in Table 2. 47 patients had invasive ductal cancer. Tumor stages were pT1 in 30 patients (56.6%), and pT2 in 23. 40 patients (75%) were node-negative; most patients (67%) were HG III. LVI and EIC were negative in more than half of the patients.

**Table 2:** Histopathologic characteristics and molecular subtypes in two groups NS: Non-significant.

		True Recurrence Group	New Primary Group	p Value
Tumor histotype	IDC	90.9% (30)	85.0% (17)	
	Other	9.1% (3)	15.0% (3)	NS
T stage	pT I	54.5% (18)	60.0% (12)	NS
N stage	pN0	69.7% (23)	85.0% (17)	NS
Grade	High grade (III)	69.7% (23)	65.0% (13)	NS
LVI	Present	51.5% (17)	65.0% (13)	NS
DCIS	Present	57.6% (19)	65.0% (13)	NS
Surgical margin width	2-5 mm	51.5% (17)	45.0% (9)	
	> 5 mm	48.5% (16)	55.0% (11)	NS
Primary tumor ER status	Positive	48.5% (16)	55.0% (11)	NS
Primary tumor PR status	Positive	18.2% ( 6)	55.0% (11)	p = 0.005
Primary tumor HER-2 status	Positive	21.2% (7)	15.0% (3)	NS
Primary tumor molecular subtypes	Luminal A	12.1% (4)	45.0% (9)	
	Luminal B	36.4% (12)	20.0% (4)	
	HER-2	21.2% (7)	15.0% (3)	
	TNBC	30.3% (10)	20.0% (4)	NS

Hormone receptor status changes were as follows: from negative to positive in 9 patients (17.0%), from positive to negative in 15 patients (28.3%) while no change in hormone receptor status was noted in the remaining 29 patients (54.7%).

Molecular subtype distribution is displayed in Table 2. HER-2 and TNBC molecular subtypes were observed more frequently in the TR group, whereas the incidence of luminal A and B subtypes were higher in the NP group. There was no statistical significance among these subgroups regarding molecular subtypes. However, PR positivity was significantly higher in the NP group (55.0% vs. 18.2%, p = 0.005).

Table 2 shows the distribution of pathologic features of tumors between the TR and NP. Despite a trend for a higher rate of pN0 (85.0% vs. 69.7%) and LVI (65.0% vs. 51.5%) in patients with NP tumors as compared to the TR group, none of these factors showed significant differences among the two subgroups. Tumor histologic type and size, HG and EIC did not significantly differ between groups. There was no significant difference among groups with regard to surgical margin width. All surgical margins were clear of tumor; more than 50% of the TR group had a margin width of 2-5 mm, 55% of the NP group had margin widths of more than 5 mm (p > 0.05).

**Figure 1:**
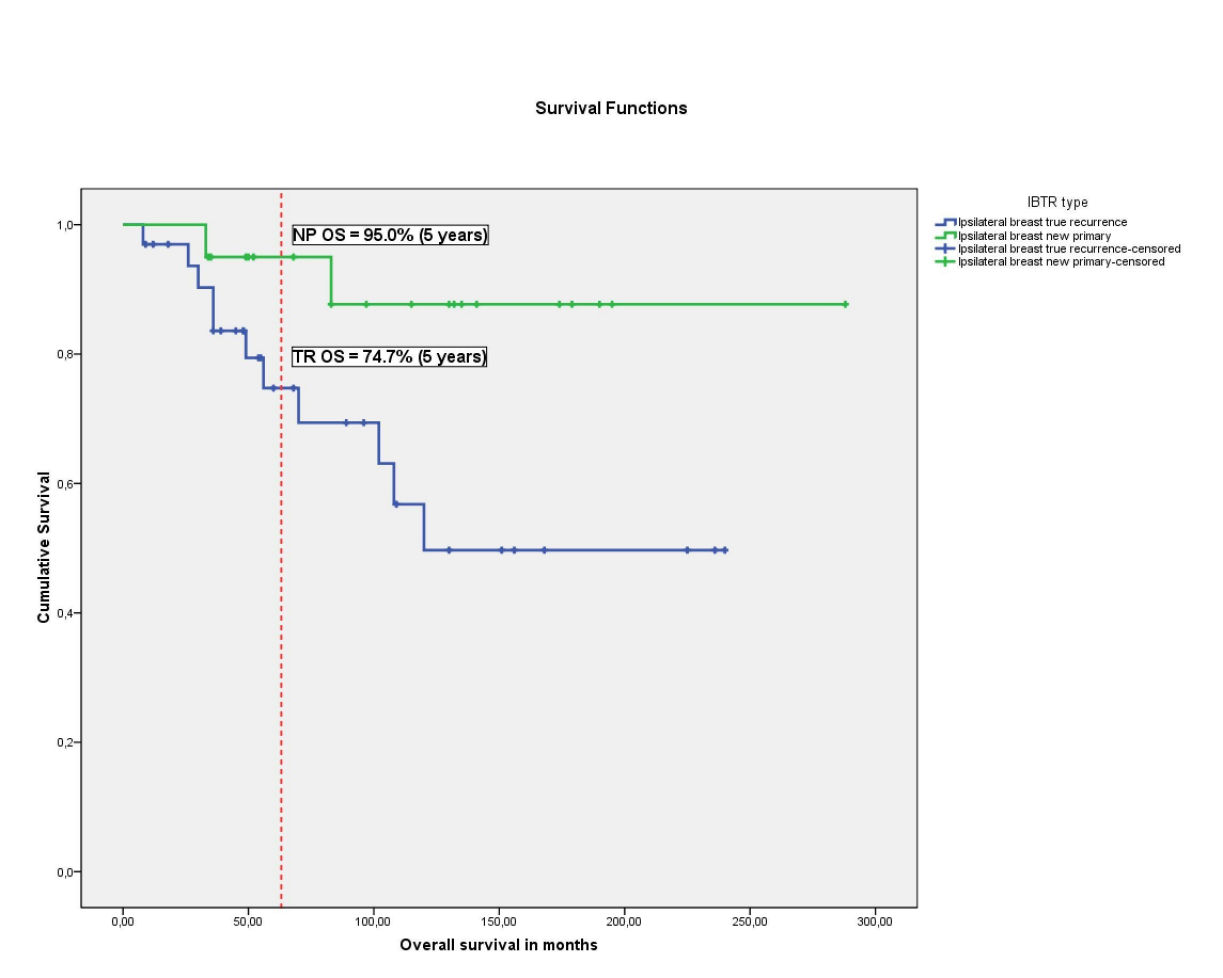
Five-year Overall Survival Rate Kaplan-Meier Survival Curves: blue curve: true local recurrences' five year overall survival; green curve: new primaries' five year overall survival.

Five-year overall survival (OS) rate for patients with ipsilateral, local recurrence was 82.8%. Overall survival was significantly higher in the NP than in the TR group (95.0% vs. 74.7% respectively), p = 0.033, Figure 1, Table 1).

Independent prognostic factors with a negative impact on OS were identified as younger age (<40 years), tumor size larger than 20 mm, high grade and triple-negative phenotype, and having a TR (HR: 4.2 (95%CI 0.98-22.76), 4.6 (95%CI 1.07-13.03), 4.0 (95%CI 0.68-46.10), 6.5 (95%CI 0.03-0.68), and 6.5 (95%CI 0.02- 0.80) respectively, p < 0.05) (Table 3).

**Table 3:** The Cox regression analysis on risk factors negatively affecting overall survival. ^a^Others include HER-2 positive group along with Luminal types A and B groups; TNBC: Triple negative breast cancer.

	Univariate Model					Multivariate Model			
	HR	95,0% CI		P		HR	95,0% CI		P
		Lower	Upper				Lower	Upper	
Age (<40 vs. >40)	1,20	0,39	3,66	0,752		4,22	0,98	22,76	0,040
Tumor size (20 mm or less vs. >20 mm)	2,04	0,67	6,26	0,211		4,58	1,07	13,03	0,032
Histologic Grade (HG III vs. HG I-II)	6,32	0,82	48,8	0,077		3,95	0,68	46,10	0,047
Luminal subtypes vs. non-luminal	2,56	0,83	7,90	0,101					
Luminal subtype (TNBC vs. others^a^)	0,33	0,11	0,98	0,046		6,49	0,03	0,68	0,011
IBTR type (True recurrence vs. New primary)	0,22	0,05	1,01	0,052		6,48	0,02	0,80	0,011

## Discussion

Long-term follow-up data from several randomized clinical studies demonstrated that breastconserving therapy in selected patients with early breast cancer had a good safety profile and had comparable survival and local/systemic recurrence rates as compared with mastectomy [3, 8, 22]. BCS is now performed more frequently due to the implementation of wide population-based mammographic screening programs, development of modern radiotherapy techniques, and the introduction of new chemotherapeutic agents [1, 23-24]. In Turkey, the ratio of patients who were treated with BCS was estimated as 35% in 2014, which could be related to the low levels of breast cancer awareness in the population and absence of a nationwide mammographic screening program resulting in detection of larger size tumors at diagnosis [25]. In our center, the overall BCS rate was determined as 68%, which places our unit among the more experienced end in this regard.

The increasing rates of BCS have highlighted the need to identify factors that have impact on local recurrence [9-10, 12, 14, 26-27]. Initial studies demonstrated that local recurrence rates after BCS were between 8-10% through 6.5-8.5 years of median follow-up [3, 21]. Recent retrospective review by van Laar C et al., and other studies showed a continuous decline in LR with reported rates in the range of 1-3% in the last decade [10, 15-17, 27]. These favorable rates could not only be attributed to improved surgical techniques and meticulous pathological evaluation, but also to improved adjuvant systemic and local management strategies. In studies that investigated patterns of local recurrences, follow-up duration and median time to ipsilateral local recurrence were reported to range between 63-80 months and 36-88 months, respectively [3, 15, 17, 26, 28-29]. Panet-Raymond et al., in their study showed that development of local recurrence occurred much earlier during follow-up in the TR group (57.6 mo) as compared to the NP group (75.6 mo) after BCS [30]. Similarly, Smith et al., reported that median time to relapse was 87.6 months for NP and 44.4 months for TR, respectively [19]. In our study, after a median follow-up period of 70 months these rates were 37.0 months (6-216) and 47.5 months (11-192) respectively. Similar to previous studies, the time to a TR was shorter in comparison to occurrence of an NP in our group of patients, despite the lack of statistical significance among the two subgroups.

The definition of ipsilateral local recurrence after BCS depends on the location and histopathologic characteristics of the tumor. In the study by Fisher et al., TR was defined as a secondary tumor that was located in the same quadrant or within 3 cm of the primary site [3]. In other studies, IBTR was considered as TR when the secondary tumor was located in the same quadrant and also carried the same histologic features as the primary tumor [17, 19]. Panet-Raymond et al., used a decision rule algorithm that exploited the tumor’s location, morphology, and molecular subtypes [30]. In our study, we adopted the St. Gallen criteria, with TR defined as ipsilateral local recurrence placed in the same quadrant as the primary tumor, showing the similar molecular phenotype [20]. New primaries were considered as tumors detected in a different quadrant with different histopathologic characteristics. In the study by Huang et al., the TR and NP rates were 62% and 38%, respectively [17]. Comparable to data from similar studies, the IBTR distribution in our study was 62.3% for TR, and 37.7% for NP [3, 17, 19-20].

In patients who developed ipsilateral local recurrences after BCS, molecular subtypes have shown significant differences between TR and NP. In previous studies where IBTR was not stratified to TR and NP subgroups, it was found that luminal B, HER2-positive phenotype, and triple-negative subtypes had higher recurrence rates than luminal A subtypes [16]. In our study, there were significantly more tumors with luminal B subtypes in the TR group as reflected by the low PR levels (Table 2). This finding is in concurrence with other studies showing that molecular phenotypes known to carry a worse prognosis are mostly found in the TR group [3, 17, 28-29]. In these studies, luminal subtypes were observed more frequently in the NP group with 77% of tumors showing hormone receptor positivity, whereas patients with HER2 positive and triple-negative breast cancer (TNBC) were more common in the TR group seen in 53% of the whole patient group [3, 17]. Our study showed a similar distribution with 65.0% of the luminal phenotypes in NP and 51.5% of HER2 and TNBC subtypes in TR. This finding is compatible with the more favorable natural history of the NP group showing a longer time to recurrence of 47.5 months, as compared to TR, which has a more proliferative tumor biology as reflected by the low PR levels and the shorter time to relapse.

Most studies have demonstrated that poor prognostic factors such as young age at diagnosis (< 40 years), high histologic grade, T2 tumor (20-50 mm), presence of LVI and EIC (>25%), and triple-negative molecular subtypes were highly correlated with worse survival in patients with TR [16, 27, 30-32]. Patients with TR were also shown to be associated with higher rates of LVI than those with NP, reported as 29.5% vs. 20.9%, respectively [15, 28-29]. Hattangadi-Gluth et al., demonstrated basal-like phenotypes were significantly associated with higher risk of local recurrence [33]. Our study also showed similar findings with higher hormone receptor positivity and lower HG rates in NP as compared to the TR group. Higher HG together with younger age at first presentation, larger tumor size, and TNBC molecular subtype were found to be associated with worse survival. We and others have shown that younger age at first diagnosis is a significant, independent risk factor affecting survival and local recurrence [33-34]. Nevertheless, our finding may be biased by the respectively higher incidence of young age at initial diagnosis observed in the Turkish population. In fact, a population-based screening program conducted by the Turkish Federation of Breast Diseases in 2014 by Ozmen et al., [25] has revealed that the percentage of patients younger than 40 was 17%, scoring being higher than the global average and being lower than only African rates (up to 30%). Consistently, in our study, 41% of patients younger than 40 is comparably higher to that reported in similar studies ranging between 16% and 27% [10, 12, 30]. Node positivity, presence of lymphovascular invasion, and extensive intraductal component had no significant correlation with outcome in our study, which may have been caused by the limited sample size. Further and larger scale studies are required to evaluate the impact of these factors on survival.

Development of ipsilateral local recurrence was related to a worse outcome in patients after BCS [35-36]. The 12-year OS rate in patients after BCS was 72%. However, a more favorable outcome was seen in patients without ipsilateral local recurrence, reported as 85% at 12 years [35]. When ipsilateral local recurrence was stratified into TR and NP groups, the overall survival rate was significantly higher in patients with NP in several studies [17-18, 34]. These figures closely reflect the results obtained in our study where new primaries' 5-year survival was 95.0% in contrast to 74.7% OS in the TR group. However, due to the higher ratio of luminal A patients in the NP group, a longer follow-up period is required to address the issue of a survival benefit, with the anticipation of further recurrences within the 5-10 year period. Furthermore, the low event rate within the molecular subtypes precluded robust statistical comparisons as the NP group had only two deaths throughout the study period, with one death seen among the TNBC and the other in the luminal B subgroups. This low number of events could be explained by the prevalence of luminal A subgroup in the NP group; whereas the TR group was enriched by luminal B, TNBC, and HER-2 subgroups, which showed an earlier occurrence of recurrences within the follow-up period. The immature follow-up time can be regarded as a major limitation of our data set.

Furthermore, not all HER-2 positive patients were treated with trastuzumab due to unavailability of this agent before 2006, making it impossible to assess the effect of anti-HER-2 treatment on IBTR. In fact, none of the three patients who developed a new primary had received trastuzumab at primary diagnosis. Nevertheless, all six patients who had received trastuzumab as part of the adjuvant systemic treatment developed a true recurrence, representing 18.2% of all TR’s. Furthermore, in our study HER 2 positivity was not identified as a significant prognostic factor for OS, as there were only 2 death events in this subgroup. In concurrence with the large datasets from adjuvant trastuzumab trials [37-39], Jia et al., reported HER-2 status to have an independent prognostic role in local recurrence as well as distant metastasis-free survival [12]. As discussed above, the small sample size and the low number of events within the distinct molecular subtypes precludes further correlative analysis and is another limitation of our study.

Triple negative phenotype was outlined as an independent prognostic factor for overall survival and true local recurrence in several studies [33, 40-42]. Similarly, our study demonstrated TNBC as a negative prognostic factor for TR and OS. This finding may lead to questioning the level of confidence when selecting BCS as a surgical method in triple negative patients. Chen et al., in their retrospective study demonstrated that BCS followed by systemic treatment was not a risk factor for tumor relapse in TN patients and should not influence the choice of surgical modality in favor of mastectomy based on histology only [40]. In concurrence with this finding, Bhoo-Pathy et al., showed that the type of surgery did not have an impact on survival in patients with TNBC; whereas adjuvant radiotherapy in patients younger than 40 was determined as the only factor that improved the outcome [43]. Since all of our patients had BCS, we could not evaluate the prognostic role of BCS in the TN subgroup. As already proven in patients with HER-2 (+) tumors, targeting the biology of the tumor is the most relevant factor that changes the natural history of the disease. For the triple negative subgroup that is in desperate need for effective treatment options, identification of potentially targetable genomic alterations remains as a challenge for the research community.

## Conclusions

Patients with a true recurrence have poorer survival as compared to those with a new primary tumor. In concurrence with previous studies, we have identified that young age less than 40, larger tumor size, high grade, and having triple negative phenotype at primary diagnosis as well as the occurrence of a TR negatively affects overall survival. These findings suggest that the role of a more aggressive adjuvant systemic therapy and irradiation should be explored further in young patients with large and high-risk tumors to prevent TR. Furthermore, a more personalized approach for an intensive follow-up program involving a thorough assessment for benefits outweighing the risks should be discussed in detail with these patients.
